# Feasibility of combination allogeneic stem cell therapy for spinal cord injury: a case report

**DOI:** 10.1186/1755-7682-3-30

**Published:** 2010-11-11

**Authors:** Thomas E Ichim, Fabio Solano, Fabian Lara, Eugenia Paris, Federico Ugalde, Jorge Paz Rodriguez, Boris Minev, Vladimir Bogin, Famela Ramos, Erik J Woods, Michael P Murphy, Amit N Patel, Robert J Harman, Neil H Riordan

**Affiliations:** 1 Medistem Inc, San Diego, USA; 2 Medistem Panama, City of Knowledge, Clayton, Panama; 3 Hospital CIMA, San Jose, Costa Rica; 4 University of California, San Diego, California, USA; 5 Cromos Pharma, Longview, Washington, USA; 6 General Biotechnology, Indianapolis, Indiana, USA; 7 Division of Medicine, Indiana University School of Medicine, Indiana, USA; 8 Dept of Cardiothoracic Surgery, University of Utah, Salt Lake City, Utah, USA; 9 Vet-Stem Inc, Poway, California, USA

## Abstract

Cellular therapy for spinal cord injury (SCI) is overviewed focusing on bone marrow mononuclear cells, olfactory ensheathing cells, and mesenchymal stem cells. A case is made for the possibility of combining cell types, as well as for allogeneic use. We report the case of 29 year old male who suffered a crush fracture of the L1 vertebral body, lacking lower sensorimotor function, being a score A on the ASIA scale. Stem cell therapy comprised of intrathecal administration of allogeneic umbilical cord blood ex-vivo expanded CD34 and umbilical cord matrix MSC was performed 5 months, 8 months, and 14 months after injury. Cell administration was well tolerated with no adverse effects observed. Neuropathic pain subsided from intermittent 10/10 to once a week 3/10 VAS. Recovery of muscle, bowel and sexual function was noted, along with a decrease in ASIA score to "D". This case supports further investigation into allogeneic-based stem cell therapies for SCI.

## Introduction

Approximately 12,000 new cases of spinal cord injury (SCI) occur per annum in the US, with about 300,000 patients living with neurological consequences [1]. Post-injury medical interventions are aimed at treatment of complications such as autonomic dysreflexia, pain, and urinary tract infections. Regenerative approaches using growth factors and various cell therapies are particularly appealing with early clinical reports of improvement using autologous bone marrow cells [2-4], olfactory ensheathing cells [5,6], and Schwann Cells [7]. In this manuscript we will describe some of the cellular/molecular aspects of spinal cord injury and regeneration, followed by overviewing selected preclinical and clinical interventions in order to provide a background for the rationale of cellular therapy for SCI. We will subsequently describe a combination approach that has yielded promising results in a case report, with the hope of stimulating further research into such allogeneic combination approaches.

### SCI Background

Nerve damage in SCI occurs in the majority of cases as a result of the combined effects of the initial physical injury, and subsequent inflammatory response caused in part by physical damage to the blood brain barrier, immune cell response to injury, and local ischemia. Typical causes of injury include contusive, compressive or stretch damage which is associated with severing of axons at the nodes of Ranvier, leading to axon retraction [8]. Furthermore, axons proximal to the area of injury that do not retract are known to develop abnormalities such as loss of myelination and swelling of the axonal body, resulting in loss of excitability [9]. Demyelination is in part believed to result from death of oligodendrocytes surrounding the axon, a process which occurs even at 3 weeks after the initial injury [10]. Importance of demyelination in this process is seen in experiments where remyelination induced by administration of Schwann cells has been demonstrated to elicit benefit in animal models of SCI [11]. Mechanistically, oligodendrocyte death appears to be related to the death receptor Fas based on: a) Pattern of expression is temporarily correlated with oligodendrocyte apoptosis in SCI models [12]; b) Genetic inactivation of Fas results in reduced oligodendrocyte death [13]; and c) Administration of soluble Fas [14] has a protective effect on SCI associated demyelination. Interestingly, administration of human umbilical cord blood stem cells in a rat SCI model results in therapeutic benefit which seems to be mediated by reduction of Fas expression [15]. Death of neurons themselves subsequent to SCI is associated with release of glutamate and other excitotoxins such as free ATP [16-18]. Interestingly, excitotoxicity occurs not only as a result of initial injury, but has also been implicated in secondary, more long-term, neuronal damage [19].

Associated with demyelination is the exposure of potassium channels which causes accumulation of the ion intraneuronally, thus further modifying ability to transmit electrical signals [20]. Inhibition of fast acting potassium channel channels using 4-aminopyridine has demonstrated some therapeutic effects in animal models of SCI [21,22], and in clinical trials [23-25].

Thus the initial injury process seems to cause: a) direct transection of neurons; b) inflammatory responses that stimulate a self-perpetuating cascade of axon retraction; c) inflammatory mediated death of oligodendrocytes; and d) stimulation of mediators such as NOGO that prevent endogenous axonal reattachement. Having described in general terms the cause of pathology, we will now overview some of the mechanisms by which the host responds to injury.

### Endogenous Regenerative Processes

Subsequent to spinal cord injuries, Schwann cells originating from the spinal root traffic to the area of injury and initiate a process of remyelinating injured axons [26]. An endogenous progenitor cell type, termed the ependymal cell, was observed in early studies to proliferate after spinal cord transection in animal models [27]. These cells, which reside in the ependyma, are known to be active in regeneration in embryonic life but their activity diminishes in adulthood [28]. A study in rats with SCI or intense exercise demonstrated BRDU incorporation into the ependymal cells under both conditions. Furthermore, the study demonstrated that ependymal cell mitosis is associated with increased proliferation and differentiation primarily into macroglia or cells with nestin phenotype [29]. It appears that ependymal cells purified from rats that underwent spinal cord injury proliferate in vitro almost 10-fold faster than ependymal cells from control animals, thus suggesting an injury-associated mitogenic event. Furthermore, in the same study it was demonstrated that transplantation of undifferentiated ependymal cells or differentiated oligodendrocyte precursor cells generated from ependymal cells, when administered to a rat model of severe spinal cord contusion induced recovery of motor activity 1 week after injury [30]. Using genetic cell fate mapping, it was demonstrated that primarily all neurogenic cells present post SCI are derived from ependymal cells, including glial cells associated with scar tissue, as well as a smaller number of oligodendrocytes [31]. Ependymal cells are known to react to exogenous growth factors, for example, intrathecal administration of EGF and FGF-2 was demonstrated to induce their proliferation [32]. Thus one therapeutic approach may be administration of exogenous factors that stimulate/accelerate natural remyelination processed. Indeed administration of FGF-2 has been demonstrated to improve locomotor function in a rat SCI model [33].

Although at a cellular level various endogenous regenerative processes may be seen in the CNS, at a functional level, post-injury regeneration is very limited. For example, after axons are severed or damaged, the myelin component of the axon is released into the extracellular environment where it generates inhibitors of neurite outgrowth [34]. Inhibitors include Nogo-A, myelin-associated glycoprotein (MAG) and oligodendrocyte myelin glycoprotein (OMgp). All three of these proteins bind to the same receptor, the Nogo-A receptor [35], and inhibit growth cone migration towards the area of injury. Inhibition of this receptor using antagonists has been demonstrated to accelerate post-SCI healing in a rat model [36].

Another inhibitor of post-injury axon repair are the reactive astrocytes that modify the ECM through secretion of factors such as chondroitin sulfate proteoglycans (CSPGs), including NG-2, neurocan, brevican, phosphacan, and versican [37,38]. These proteoglycans, specifically their side chains, are known to inhibit nerve growth and in some cases contribute to formation of the glial scar that serves as a physical barrier to axon regrowth [39]. Administration of the enzyme chondroitinase ABC, which cleaves these side chains has been demonstrated to reduce axon-inhibitory activity of CSPGs in vitro [40]. In vivo studies demonstrated that condroitinase ABC therapy accelerates recovery in animal models of SCI [41]. Supporting the hypothesis that chondroitinase ABC benefits on SCI are mediated by "inhibiting the inhibitor" of neurite outgrowth, a recent study demonstrated that treatment with the enzyme provides a therapeutic window in which rehabilitation programs function optimally [42].

Angiogenesis is an integral part of numerous healing processes. In the context of spinal cord injury, hypoxia inducible factor (HIF)-1 alpha activates numerous downstream effectors such as BDNF, VEGF, SDF-1, TrkB, Nrp-1, CXCR4 and NO, that attempt to restore the "neurovascular niche" after damage has occurred [43]. These molecules act not only on creation of new vasculature but also are involved in neurogenesis. The critical link between neural recovery and angiogenesis may be seen in animal models of post-stroke regeneration where cord blood derived cells appear to elicit effects primarily by stimulating de novo vasculature which causes expansion of endogenous progenitors [44]. Transfection of neuronal progenitors with the angiogenic factor VEGF has been shown to increase angiogenesis and recovery [45]. Additionally, administration of human CD133 peripheral blood progenitor cells accelerates post-injury healing in part through secretion of VEGF [46]. There is some evidence that counter-angiogenic mechanisms are present in the late post-injury setting. For example Mueller et al showed that approximately 7 days post injury an accumulation of endostatin/collagen XVIII is observed in the areas associated with vascular remodeling [47].

Thus several endogenous repair mechanisms exist including activation of ependymal cells and generation of oligodendrocyte progeny, and angiogenesis. These are inhibited in part by production of various agents such as NOGO and ECM degradation productions. Tipping the balance in favor of regeneration by exogenous growth factor administration or providing inhibitors of inhibitors is a promising approach. By understanding the background biological post-injury terrain administration of exogenous stem cells may be used for optimal results.

### Stem Cell Therapy for SCI

#### Olfactory Ensheathing Cells

Given an endogenous reparative component, albeit mild, exists in the injured CNS, an aim of research is to augment this process. Initial work in the 1980s focused on providing a "bridge" for axon growth across the scar tissue formed as a result of injury. Aguayo et al placed autologous sciatic nerve grafts between the lower cervical/upper thoracic spinal cord and the medulla oblongata in injured mice and rats. At 1-7 months after grafting, microscopic studies demonstrated myelinated axons had migrated and grown across the graft. Horseradish peroxidase was injected intraxonally to demonstrate functional integrity of the axons [48]. Electrophysiological improvements after excision of the spinal cord dorsal columns was noted 5-6 months after application of peripheral nerve graft across the injured area [49]. Work on using grafted cells led to interest in olfactory ensheathing cells as a potential source of glial cells for transplantation. These cells function on the one hand to physically wrap up numerous axons to form large bundles of axons, and on the other hand are known to produce high levels of axon-regenerating growth factors [50,51]. Olfactory ensheathing cells have the unique property of being able to repeatedly migrate from the nasal olfactory mucosa, which is part of the peripheral nervous system into the central nervous system environment of the olfactory bulb [52]. This is in contrast to Schwann cells which are much slower at integrating into the central nervous system. Several studies have shown that combinations of olfactory ensheathing cells with Schwann cells causes additive therapeutic effects [53-55].

Purification of olfactory ensheathing cells can be performed by selection for cells expressing the O4 antigen but lacking expression of galactocerebroside. These cells appear to have a unique phenotype in contrast to other glial cells or Schwann cells, for example, they have astrocyte markers and lack a basal lamina and collagen fibrils [56,57]. Administration of olfactory ensheathing cells across transected spinal cord in several models has resulted in axonal regeneration and restoration of conduction velocity [58-60].

Clinical implementation of olfactory ensheathing cells for SCI has been reported in several trials. Lima et al treated 7 patients with ASIA class A traumatic-induced SCI from C4-T6. All patients reported improvement in ASIA motor scores, with 2 patient reporting return of sensation to bladder and one gaining control of anal sphincter. The therapy was well tolerated, however adverse effects included a sensory decrease in one patient [5]. A subsequent study by Mackay-Sim et al [6] reported no major benefit in a 3 year follow-up of patients with traumatic injury to the thoracic spine (T4-T10) that occurred 6-36 months prior to therapeutic intervention. Three patients were administered ex vivo expanded autologous olfactory ensheath cells, and compared to 3 control patients. All patients had a sustained and complete loss of sensory and motor function below the injury, being classified as ASIA Category A. Cells were administered into the damaged area of the spinal cord, as well as at the proximal and distal ends of the intact cord subsequent to laminectomy and durotomy. No improvement was observed in functional parameters tested including ASIA motor and sensory assessment, COVS, or FIMS. Radiological assessment was unremarkable in the treated patients, indicating safety of the procedure. One treated patient had an increased sensitivity to light touch that was observed over 3 segments.

#### Schwann Cells

Schwann cells are terminally differentiated cells of the peripheral nervous system whose main function is remyelination and promoting axonal regeneration. These cells have been used experimentally since 1981 for the purpose of accelerating healing post SCI [61]. Since then, numerous animal studies have been conducted. In a comprehensive review, Tetzlaff et al [62] discussed 35 rodent studies in which the overall findings where that Schwann cells possessed ability to regenerate sensory axons from the dorsal root ganglia and propriospinal axons adjacent to the injury site. However the cells were incapable of healing brainstem spinal axons, nor where they able to cause axons exit and reenter the host spinal cord. Functionally, benefits in locomotion, and neurological parameters subsequent to Schwann cell administration have been noted in SCI induced by subacute contusion [63], photochemical damage [64], and transection [65].

#### Schwann cell clinical trial

Schwann cells are attractive from a clinical perspective because of the possibility of using autologous cells, thus avoiding allogeneic immunological issues, or ethical dilemmas associated with material of fetal origin. Saberi et al [7] reported preliminary results in 4 patients treated with autologous Schwann cells suffering from chronic thoracic SCI. Schwann cells were isolated from the sural nerve and grown in vitro without passaging. They were injected into at a concentration of 3-4.5 million cells in a total volume of 300 uL into the injured segment of the cord adjacent to the rostral and caudal ends.

No adverse effects or functional improvements were noted, nor was MRI capable of identifying transplanted cells. One of four patients reported increased motor and sensory improvement after treatment.

#### Bone marrow stem cells

Bone marrow mononuclear cells have been classically used as a hematopoietic stem cell source for bone marrow transplantation, however some efficacy has been demonstrated in accelerating healing in cardiac [66], hepatic [67,68], and vascular injury [69,70]. Given the bone marrow contains cells capable of providing trophic support for neurons [71-74], as well as cells possibly capable of directly differentiating into neurons [75,76], a series of investigations have been performed in this area. In animal models it has been demonstrated that bone marrow mononuclear cells [74], CD34 hematopoietic stem cells [77], mesenchymal stem cells [78-80], and in vitro differentiated mesenchymal stem cells [81], all possess some level of SCI regenerative activity.

The dog is a very relevant large animal model of SCI. In a comprehensive, blinded study of spinal cord compressive injury in the dog, Jung et al. demonstrated a biologically and statistically improved outcome with therapy using autologous and allogeneic bone marrow mesenchymal stem cells. MRI, histology, and immunofluorescence supported the direct effect of the therapy on repair of the SCI [79].

Administration of bone marrow mononuclear cells via lumbar puncture in patients with spinal cord injury has been demonstrated to induce no serious adverse effects [82]. A study of 8 patients with chronic and acute SCI reported administration of bone marrow mononuclear cells via intravenous route as well as into the spinal canal and directly into the spinal cord. The authors observed improvement in bladder function, as well as benefit using the ASIA, Barthel (quality of life), Frankel, and Ashworth instruments. Furthermore, it was stated that 52 SCI patients have been treated with no serious adverse events [2]. Another study examined 20 SCI patients complete injury who were administered autologous bone marrow mononuclear cells in an acute (10-30 days after injury) and chronic (2-17 months after injury) setting. Improvement in motor and/or sensory functions was observed within 3 months in 5 of the 7 acute patients, and in 1 of 13 chronic patients. No adverse effects were reported with 11 patients being followed up for more than 2 years post stem cell administration [3]. Thus it appears that autologous bone marrow cells have a favorable safety profile, with some signal of efficacy, although larger studies are required.

These approaches promoted a more aggressive protocol combining stem cell administration into the area of injury, together with endogenous stem cell mobilization. Yoon et al [4] assessed a total of 48 patients having complete ASIA A SCI at the cervical or thoracic area that were either a) untreated; b) treated 2 weeks or less after the injury (acute); c) treated 2-8 weeks after the injury (subacute); or d) treated more than 8 weeks after injury (chronic). Treatment consisted of 10^8 ^autologous bone marrow mononuclear cells administered in six injections of 300-uL surrounding the lesion site with the injection depth of 5 mm from the dorsal surface and 5 mm lateral from the midline. The lesion was exposed by laminectomy one vertebra above to one below and the dura mater was then incised, sparing the arachnoid, which was subsequently opened separately with microscissors. GM-CSF was administered in 5 monthly cycles of 5 daily injections at the beginning of the month at a concentration of 250 g/m^2 ^of body surface area. Injection procedure was uneventful, with adverse events being mild, typically consistent with GM-CSF administration. An increased incidence of neuropathic pain was observed in the subacute and chronically treated patients as compared to acute and control patients. Neurological improvement (AIS A to AIS B or AIS C) was observed in 29.5% and 33.3% of patients in the acute and sub-acute groups, respectively. No improvement was noted in the chronic group, 7.7% and 12.5% was observed in the control, and a historical control [83], respectively. Changes in spinal diameter, both increases and decreases occurred in the treated groups as compared to untreated. Functional MRI studies indicated regeneration of functional neural pathways in some of the treated patients. Interestingly, a correlation between response and GM-CSF induced leukocytosis was observed. This study is a continuation of previous work by the same group, Park et al. [84], in which 6 patients with complete AIS grade A SCI were treated with an identical protocol. Four of the patients went from AIS A to C, one patient when from AIS A to B, and one had no change.

#### Adipose-derived Stem and Progenitor Cells

Mesenchymal stem cells derived from adipose tissue have been extensively described in the literature, including significant support for the ability of these progenitors to differentiate into many neural cell types [85-87]. In a similar experiment to the canine SCI bone manuscript described above [79], Ryu and et al [88], conducted a blinded, placebo controlled canine clinical study of SCI using and cultured allogeneic adipose stem cells in a model of acute SCI with cells administered intralesionally one week after SCI. The treated groups both statistically outperformed the saline control group and showed significant clinical and histological improvement in ambulation and cord neural repair.

#### Cord Blood/Placental Derived Cells

Umbilical cord and Wharton's jelly derived MSC offer unique therapeutic characteristics in comparison to bone marrow MSC. Specifically, longer telomeres, increased passage ability without loss of differentiation potential, and more potent cytokine release activity are some attractive features of this cell population [89]. Yang et al [90], generated a population of Wharton's jelly derived MSC and administered the cells alone or after treatment with neural conditioned media for 3 or 6 days into immunocompetent rats subsequent to complete spinal cord transection. Improvements in locomotion were observed in animals receiving MSC or MSC treated with conditioned media. Regeneration of corticospinal tract axons and neurofilament-positive fibers was observed. Mechanistically, the cells appeared to function at least in part by production of growth factors such as bFGF, GITR, VEGFR3, neurotrophin-3, and NAP-2. Studies are currently underway using combinations of factors such as BDNF together with cord MSC to augment regenerative activity post-SCI [91]. Clinically, a case report from Korea describes the administration of multipotent cord blood derived stem cells into a SCI patient by local injection. These cells elicited improvement in ability to move hips and thighs, as well as augmented sensory activity 41 days after cell therapy. Radiologically documented regeneration of spinal cord and cauda equina was noted [92].

Cord blood derived cells have been described to stimulate post-infarct neurogenesis through stimulation of angiogenesis [44], preclinical studies have sought to determine whether this may be replicated in conditions of spinal cord injury. Using a rat left spinal cord hemisection model, Zhao et al demonstrated superiority functional recovery according to the Tarlov score by intraspinal administration of human CD34 cells derived from cord blood versus bone marrow [93]. Both cell populations where shown to survive and migrate into the area of injury, as well as differentiate into glial (GFAP+) or neural (NeuN+)-like cells. Purified CD34 cells from cord blood were demonstrated in another study to augment functional recovery as assessed by the Basso-Beattie-Bresnahan Locomotor Scale, reduce the area of the cystic cavity at the site of injury, increase white matter volume, and stimulate axonal regeneration [94]. Mechanistically it appears that cord blood CD34 cells mediate effects in part through secretion of glial cell line-derived neurotrophic factor (GDNF) and vascular endothelial growth factor (VEGF) [95].

#### Fetal/ES Derived Neural Progenitors

Fetal-derived neurons have been shown to survive, differentiate and integrate into the host spinal cord after injury [96]. When used together with scaffolds or ventral root implants, these cells can grow their axons along the whole length of the peripheral nerves to reach muscles in the limb and restore function after transection [97]. In addition to local placement of fetal neurons in the damaged area, systemic administration of fetal neural precursors results in local homing through a SDF-1 and HGF-1-dependent mechanism [98]. Although numerous experiments have demonstrated varying degrees of efficacy in animal models [99-103], the risk of oncogenesis raises concerns for clinical testing. These fears where increased when an ataxia telangiectasia patient receiving 8-12 week old human fetal neuron preparations developed a multi-focal brain tumor containing donor cell karyotype after transplantation [104]. Another concern has been development of allodynia as a result of improper nervous connections being made [105]. Embryonic stem (ES) cells offer the ability to generate specific nervous system cells useful for addressing various aspects of the SCI process. For example, ES generated neural precursors [106], motor neurons [107], and oligodendrocytes [108,109] have all been used to induce amelioration of SCI in animal models. Recently Geron Inc received an FDA approval to initiate clinical trials using ES-derived oligodendrocytes in SCI [110], which was subsequently placed on clinical hold before patient treatment occurred [111]. At present ES-based approaches are limited by similar concerns as fetal stem cell based approaches in terms of oncogenesis and allodynia.

## Case Report: Informed Consent

Before administration of experimental intervention, the patient signed an informed consent form in which the experimental nature of the procedure to be performed was explained in detail. Additionally the patient was made aware of possible adverse events of the procedure, including, but not limited to, increases in neuropathic pain, possibility of ectopic tissue formation, and uncertainty whether benefits will be obtained by the procedure. The protocol was approved by the local Institutional Review Board.

## Case Report: Combination of Placental MSC and Cord CD34

Currently stem cell clinical trials in SCI have been focused on use of autologous bone marrow, MSC, or olfactory ensheathing cells, with one case report of allogeneic cord derived multipotent progenitor cell [92]. The possibility of using allogeneic stem cell sources would allow for generation of standardized, "ready to use" cellular productions that could be widely implemented. While allogeneic MSC have been used for late stage clinical trials with safety being established [112], little work has been reported on allogeneic CD34 cells in absence of myeloablation/immune suppression. The authors have recently published a series of 114 patients with neurodegenerative conditions treated with allogeneic non-matched cord blood cells. While no adverse events were associated with therapy, little is known about potential efficacy of this approach [113]. The possibility of a combination approach would be conceptually appealing given that MSC are known anti-inflammatory and growth factor producers, whereas CD34 cells produce angiogenic factors and in some cases have been demonstrated to differentiate into neurons directly. Here we describe a protocol based on a combination of intrathecal administration of CD34 and placental derived MSC.

The patient was born on November 5, 1979 and suffered a spinal cord injury in a single propeller engine airplane crash on May 13^th ^of 2008. At the time of the accident he was diagnosed with an incomplete spinal cord injury at the level T12 - L1, and crush fracture of the L1 vertebral body which was described as a type A in the ASIA scale. The patient was initially treated at Hospital Mexico in Costa Rica on the day of the accident. The spine was stabilized using paravertebral rods from T11 to L2. Bone fragments were removed from the spinal canal. After 1 week of being hospitalized, he was transferred to the National Rehabilitation Center in Costa Rica, where he remained for 4 weeks. He was required to use a harness for lumbar support and had to remain in the supine position and physical therapy focused on stretching exercises. Neuropathic pain was present at a 10/10 for which he was administered Lyrica at 300 mg/day.

Cellular treatment was performed in 3 cycles between Oct 31-Nov 20, 2008, Jan 21-30, 2009, and July 1-10, 2009 consisting of intrathecal administration of CD34 and MSC, with the last cycle also receiving IV injections (Table 1). MSC and CD34 cells where isolated from the placental matrix and cord blood, respectively, as previously described by us [114]. No adverse effects were associated with the lumbar puncture procedure, nor were immunological reactions or GVHD noted. A progressive improvement in muscle strength was noted during the observation period, with last evaluation performed in January of 2010 (Table 2). Additionally, increased sensation in various dermatomes was noted as shown in Table 3. As of January 7^th^, 2010 he is categorized as an ASIA D patient. He recovered urologic, sexual and bowel function. The patient discontinued use of Lyrica and reports occasional pain once a week at a level of 3/10. Throughout the courses of cell treatments the patient received physical therapy.

**Table 1:** Administration Schedule

Date	Route	CD34	MSC
*Cycle 1*			

Oct 31 2008	IT	3 million	6 million

Nov 11 2008	IT	3 million	6 million

Nov 14 2008	IT	3 million	6 million

Nov 18 2008	IT	3 million	6 million

Nov 20 2008	IT	3 million	6 million

			

*Cycle 2*			

Jan 21 2009	IT	3 million	6 million

Jan 23 2009	IT	3 million	6 million

Jan 26 2009	IT	3 million	7 million

Jan 28 2009	IT	3 million	7 million

Jan 30 2009	IT	3 million	7 million

			

*Cycle 3*			

Jul 1 2009	IT	1.5 million	5.7 million

Jul 3 2009	IT	1.5 million	6.3 million

July 6 2009	IT	1.5 million	6.3 million

	IV	1.5 million	3.9 million

July 8 2009	IT	1.5 million	6.12 million

	IV	1.5 million	3.9 million

July 10 2009	IT	1.5 million	6.12 million

**Table 2:** Muscle Strength evaluation by Groups*

	Jun 16 2008		Nov 13 2008		Feb 23 2009		Aug 5 2009		Jan 7 2010	
	**Right**	**Left**	**Right**	**Left**	**Right**	**Left**	**Right**	**Left**	**Right**	**Left**

**Hips**:										

Flexors	1+	2	2	4	2+	5-	3-	5	3	5

Extensors	1+	2	2	4	2	5	2+	5	3-	5

Abductors	1+	2	2	4-	2-	5	2	5	4	5

Adductors	1+	3	2	4	2	5-	2+	5	4	5

Internal Rotators	1+	2-	1	4-	1+	5-	3-	5	3	5

External Rotators	1	2	1	3+	2-	5-	3-	5	3	5

**Knee**:										

Flexors	1	2+	1	4	1+	4-	2	5-	3-	5

Extensors	1	4-	1	4-	2-	5	2	5	4	5

**Ankle**:										

Dorsiflexors	0	4-	1-	4-	1	5	1-	5	1	5

Plantaflexors	1-	3-	3	4	3+	5	3+	5	3	5

Eversion	0	3-	1	4-	1	5	1+	5	2	5

Inversion	0	3-	1	4-	1	5	1+	5	2	5

**Toes**:										

Flexors	0	4-	1	3+	1	5	2-	5	1	5

Extensors	0	4-	0	3+	1-	5	1+	5	1	5

*Spinal cord injury occurred May 13, 2008. Patient received stem cell therapy Oct 31-Nov 20, 2008, Jan 21-Jan 30, 2009, and July 1-10, 2009.

**Table 3:** Sensation by Dermatome*

	June 16 2008		Feb 23 2009		Sept 10 2009	
	**Right**	**Left**	**Right**	**Left**	**Right**	**Left**

T10	2	2	2	2	2	2

T11	1	1	1	2	1	2

T12	0	1	1	2	1	2

L1	0	0	1	2	1	2

L2	0	1	1	1	1	1

L3	0	0	1	1	1	1

L4	0	1	1	2	1	2

L5	0	0	1	2	1	2

S1	0	0	1	2	2	2

S2	0	0	1	2	2	2

*Spinal cord injury occurred May 13, 2008. Patient received stem cell therapy Oct 31-Nov 20, 2008, Jan 21-Jan 30, 2009, and July 1-10, 2009.

Spontaneous recovery of spinal cord injury patients has been previously reported in the literature [115], which is a concern for clinical trial design in this area [116]. However, recovery of bowel and sexual function in a patient presenting with ASIA A injury is extremely rare. Given that this study is a case report, we are cautious in the interpretation of efficacy data. However, the demonstration of feasibility of intrathecal combination stem cell administration, without occurrence of neuropathic pain or ectopic tissue formation supports further investigation of this approach in a standardized manner.

## Competing interests

TEI, VB, and NHR are shareholders and management of the biotechnology company Medistem Inc, which has patent applications and a filed IND on the endometrial regenerative cells.

## Authors' contributions

TEI, FS, FL, EP, FU, JPR, BM, VB, FR, EJW, MPM, ANP, RJH, NHR were involved in writing of the manuscript, literature review, and assessment of patient data. FS, FL, EP, and JPR were involved in patient care. All authors read and approved the final manuscript.
